# Novel Graph-Based Model With Biaffine Attention for Family History Extraction From Clinical Text: Modeling Study

**DOI:** 10.2196/23587

**Published:** 2021-04-21

**Authors:** Kecheng Zhan, Weihua Peng, Ying Xiong, Huhao Fu, Qingcai Chen, Xiaolong Wang, Buzhou Tang

**Affiliations:** 1 Key Laboratory of Network Oriented Intelligent Computation Harbin Institute of Technology Shenzhen China; 2 Baidu International Technology (Shenzhen) Co, Ltd Shenzhen China; 3 Peng Cheng Laboratory Shenzhen China

**Keywords:** family history information, named entity recognition, relation extraction, deep biaffine attention

## Abstract

**Background:**

Family history information, including information on family members, side of the family of family members, living status of family members, and observations of family members, plays an important role in disease diagnosis and treatment. Family member information extraction aims to extract family history information from semistructured/unstructured text in electronic health records (EHRs), which is a challenging task regarding named entity recognition (NER) and relation extraction (RE), where named entities refer to family members, living status, and observations, and relations refer to relations between family members and living status, and relations between family members and observations.

**Objective:**

This study aimed to introduce the system we developed for the 2019 n2c2/OHNLP track on family history extraction, which can jointly extract entities and relations about family history information from clinical text.

**Methods:**

We proposed a novel graph-based model with biaffine attention for family history extraction from clinical text. In this model, we first designed a graph to represent family history information, that is, representing NER and RE regarding family history in a unified way, and then introduced a biaffine attention mechanism to extract family history information in clinical text. Convolution neural network (CNN)-Bidirectional Long Short Term Memory network (BiLSTM) and Bidirectional Encoder Representation from Transformers (BERT) were used to encode the input sentence, and a biaffine classifier was used to extract family history information. In addition, we developed a postprocessing module to adjust the results. A system based on the proposed method was developed for the 2019 n2c2/OHNLP shared task track on family history information extraction.

**Results:**

Our system ranked first in the challenge, and the F1 scores of the best system on the NER subtask and RE subtask were 0.8745 and 0.6810, respectively. After the challenge, we further fine tuned the parameters and improved the F1 scores of the two subtasks to 0.8823 and 0.7048, respectively.

**Conclusions:**

The experimental results showed that the system based on the proposed method can extract family history information from clinical text effectively.

## Introduction

Family history information plays an important role in the diagnosis and treatment of diseases, especially genetic disorders. Family history information is always embedded in electronic health records (EHRs) in a semistructured/unstructured format, which needs to be unlocked by natural language processing (NLP) technology.

In order to promote research on family history information extraction, Harvard Medical School and Mayo Clinic organized national NLP challenges on family history information extraction in 2018 and 2019. The family history information extraction task includes the following two subtasks: (1) recognizing family members, living status, and observations and (2) determining which family members the recognized living status and observations belong to, which correspond to two fundamental NLP tasks, namely named entity recognition (NER) and relation extraction (RE). The NER task is usually regarded as a sequence labeling task, while the RE task is the subsequent classification task, and they are tackled by pipeline methods.

For the NER task, traditional machine learning methods, such as hidden Markov model (HMM), conditional random field (CRF) [1], and structured support vector machine (SSVM) [2], and deep learning methods, such as Bidirectional Long Short Term Memory network (BiLSTM) CRF [3] and its variants [4,5], have been widely applied. For the RE task, the typical machine learning methods include support vector machine (SVM) [6], convolutional neural network (CNN) [7], and recurrent neural network [8]. The methods mentioned above have also been applied for clinical entity recognition and RE, such as the NLP challenges organized by i2b2 in 2009 [9], 2010 [10], 2012 [11], and 2014 [12], the NLP challenges organized by SemEval in 2015 [13] and 2016 [14], the NLP challenges organized by ShARe/CLEF in 2013 [15] and 2014 [16], and the NLP challenges organized by BioCreative/OHNLP in 2018 [17]. Most of these methods process NER and RE tasks in a pipeline way, which can suffer from error propagation [18].

A number of joint learning methods have been proposed [18,19] for NER and RE subtasks to avoid error propagation from NER to RE. In the case of family history information extraction, Shi et al [17] developed deep joint learning based on the BiLSTM that won the 2018 BioCreative/OHNLP challenge [20]. Joint learning methods generally used pretrained neural language models. Neural language models pretrained on large-scale unlabeled text have recently been proven to be surprisingly effective in many downstream tasks, and Bidirectional Encoder Representation from Transformers (BERT) [21] is one of the most popular neural language models.

In this study, we proposed a novel graph-based model with biaffine attention. Inspired by the dependency parsing task [22,23], we designed a novel graph-based schema to represent family history information and introduced deep biaffine attention [22,23] to extract family history information from clinical text. A system based on the proposed method was developed for the 2019 n2c2/OHNLP challenge on family history information extraction, and it achieved the highest F1 scores of 0.8823 on subtask1 and 0.7048 on subtask2.

## Methods

### Task Description

There were two subtasks in the 2019 n2c2/OHNLP challenge on family history information extraction. For subtask1, we need to recognize family members with the side of the family, living status mentioned in clinical text, and observations in the family history. All family members can be normalized to standard forms in Table 1. The property of family members named “side of family” includes the following three possible values: NA (“not applicable”), maternal, and paternal. Following the work of Shi et al [17], we compared two different strategies. The first strategy recognized three types of entities (family member, observation, and living status) and determined the “side of family” property for each family member entity through a postprocessing module. The second strategy recognized five types of entities (NA, maternal, paternal, observation, and living status), directly determining the “side of family” property of family members.

For subtask2, we need to extract the relations between family members, observations, and living status. Living status is used to represent the health status of family members, and it has the two properties of “Alive” and “Healthy.” Each property was measured by a real-valued score (yes: 2, NA: 1, and no: 0). The total living status score of family members was their alive score multiplied by their health score. We also need to predict the negation information (Negated and Non_Negated) for each observation, that is, to judge whether the family members have certain diseases or not.

**Table 1:** Normalized family member names.

Degree	Normalized family member names
1	Father, Mother, Parent, Sister, Brother, Daughter, Son, and Child
2	Grandmother, Grandfather, Grandparent, Cousin, Sibling, Aunt, and Uncle

### Data Statistics

We conducted experiments on the corpus provided by the 2018 and 2019 n2c2/OHNLP shared task tracks on family history information extraction. The training set of the 2019 n2c2/OHNLP shared task together with the test set of the 2018 BioCreative/OHNLP shared task was used as the final training set of 149 EHRs for model training. The test data set of the 2019 n2c2/OHNLP shared task, including 117 EHRs, was used for the model test. During model training, we randomly selected a development set of 14 EHRs from the training set for parameter optimization. The statistics of the corpus used in this study is shown in Table 2.

**Table 2:** Detailed data set statistics.

Item	Training set, n	Development set, n	Test set, n
Document	149	14	117
Sentence	770	71	644
FM^a^: overall	1128	94	—^b^
FM: NA^c^	631	55	—
FM: maternal	272	24	—
FM: paternal	225	15	—
OB^d^	1439	127	—
LS^e^	596	52	—
FM-OB: overall	1064	97	—
FM-OB: NA-OB	575	57	—
FM-OB: maternal-OB	265	23	—
FM-OB: paternal-OB	224	17	—
FM-LS: overall	605	53	—
FM-LS: NA-LS	334	29	—
FM-LS: maternal-LS	145	12	—
FM-LS: paternal-LS	126	12	—

^a^FM: family member.

^b^Not available.

^c^NA: not applicable.

^d^OB: observation.

^e^LS: living status.

### Graph-Based Schema

**Figure 1:**
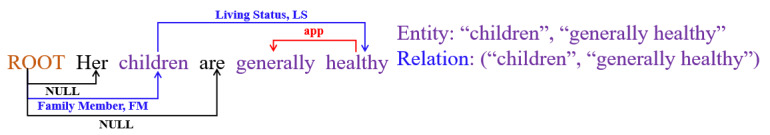
Example of using a graph-based schema to represent family history information.

Similar to the dependency parsing task where each token has a head token, we transformed the family history information extraction task to a dependency parsing problem, where a dummy root (denoted by “ROOT”) was appended to each sentence at the beginning and arcs denoted links between two tokens. In the “dependency parsing tree” of a sentence, tokens in each entity were connected together by an “app” arc from right to left, two entities with a relation were connected through linking the right most token by an arc labeled with the entity type, and tokens not in any entity were connected with the “ROOT” node by “NULL” arcs. Figure 1 shows an example of using a “dependency parsing tree” to represent family history information extraction, where the family member entity “children” was determined by the “Family Member” arc from “ROOT” to “children,” the living status entity “generally healthy” was determined by “generally 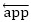 generally,” and the relation between “children” and “generally healthy” was determined by the arc from “children” to “healthy” 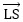.

### Model Architecture

**Figure 2:**
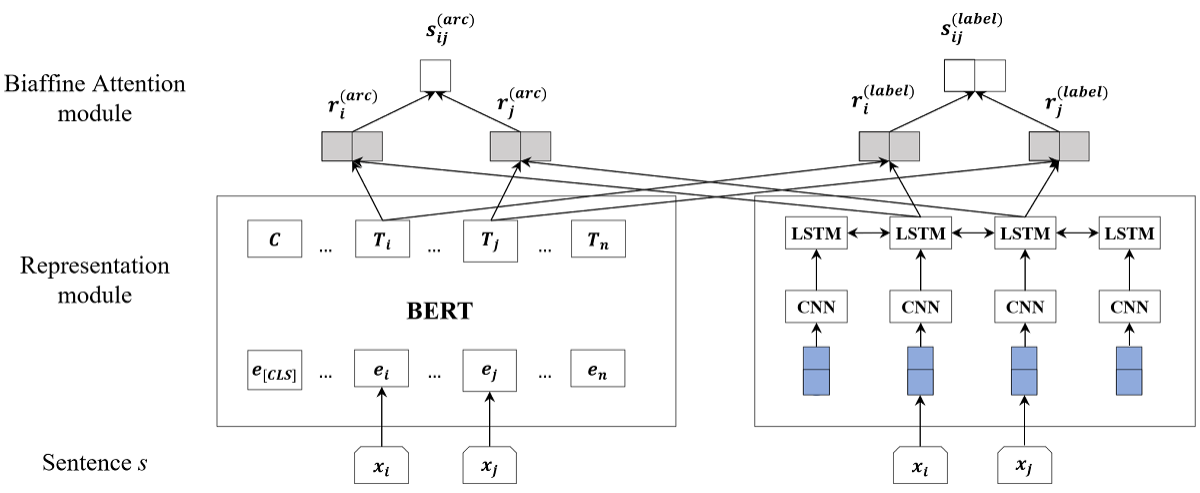
Overview architecture of our model.

As shown in Figure 2, our model contained the following two main parts: (1) a representation module, which represented input text using BERT and CNN-BiLSTM and (2) a biaffine attention module to predict label score vectors, including unlabeled arc prediction (top left in Figure 2) and arc label prediction (top right in Figure 2). We have presented them in the following sections in detail.

### Representation Layer

Given a sentence *s* = *x*_1_…*x_i_*…*x_n_*, where *x_i_* is the *i*th token of *s*, we used BERT and CNN-BiLSTM to represent it separately as follows:

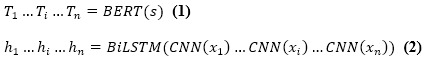



where CNN [4] is first used to get the character-level representation of each token, and BiLSTM is then used to get the contextual representation of each token in CNN-BiLSTM. The final representation of token *x_i_* is

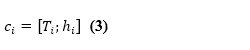



### Biaffine Attention Layer

#### Unlabeled Arc Prediction

Considering the *i*th token and the *j*th token, we fed their corresponding representations into a bilinear transformation extension called a biaffine function to get the score of the arc from token *i* (head) to *j* (dependent) as follows:

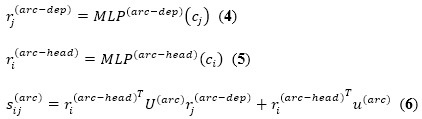



where *r_j_*^(^*^arc^*^−^*^dep^*^)^∈R*^p^* and *r_j_*^(^*^arc^*^−^*^head^*^)^∈R*^p^* are the outputs of multilayer perceptron, *U*^(^*^arc^*^)^∈R*^p^*^×^*^p^* is a weight matrix controlling the strength of the arc from token *i* to *j*, and *u*^(^*^arc^*^)^∈R*^p^* is a bias vector.

Assume that *s_j_*^(^*^arc^*^)^ = [*s*_1_*_j_*^(^*^arc^*^)^;…;s*_nj_*^(^*^arc^*^)^] is the score vector of all possible heads of the *j*th token. We adopted the softmax function to compute the probability distribution *d_j_* of all possible heads of token *j* and the cross-entropy between the predicted *d_j_* and gold standard *d_j_*^(^*^arc^*^)^ as the loss function as follows:

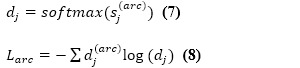



Thereafter, the best head of token *j* was determined according to

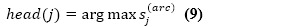



#### Arc Label Prediction

For each unlabeled arc, we need to determine its label. Assume that *s_ij_*^(^*^lab^*^)^∈R^|^*^L^*^|^ is the label score vector for each arc from token *i* to *j*, where |*L*| is the size of the label set. We can compute *s_ij_*^(^*^lab^*^)^ as follows:

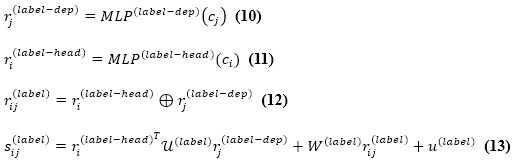



where *r_j_*^(^*^label^*^−^*^dep^*^)^∈R^|^*^L^*^|×^*^p^* and *r_j_*^(^*^label^*^−^*^head^*^)^∈R^|^*^L^*^|×^*^p^* are outputs of the multilayer perceptron, *U*^(^*^label^*^)^∈R^|^*^L^*^|×^*^p^*^×^*^p^* is a third-order tensor, *W*^(^*^label^*^)^∈R^|^*^L^*^|×2^*^p^* is a weight matrix, and *u*^(^*^label^*^)^∈R^|^*^L^*^|^ is a bias vector.

We also adopted the softmax function to compute the probability distribution *d_ij_* of all possible labels of the arc from token *i* to *j* and the cross-entropy between the predicted *d_ij_* and gold standard *d_ij_*^(^*^label^*^)^ as the loss function as follows:

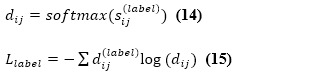



Thereafter, the best label of the arc from token *i* to *j* was determined by

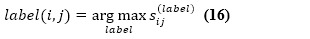



The total loss function was set as

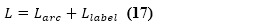



### Postprocessing Rules

We designed a rule-based postprocessing module to adjust the outputs of our model. It included the following five parts:

1. Converting the output to entities and relations.

(1) Combining all tokens connected by “app” arcs to form entities and assigning them the label of their last token.

(2) If there was an arc between two entities, but not an “app” arc, there was a relation between them.

2. Normalizing family members.

(1) Converting family member entities into normalized forms as shown in Table 1. For example, we converted the recognized “father’s father” into “grandfather” and “aunt’s son” into “cousin.”

(2) Excluding unnecessary family members. For example, a patient’s nonblood relatives, such as “father” in section “partner’s father,” should be removed. If the family member “father” belonged to section “partner’s father,” we removed “father” since father-in-law was not in Table 1.

3. Determining the side of family members when using the strategy of three types of entities.

(1) If a family member was a first-degree relative, the side of the family was set as “NA.”

(2) If a family member was in the section “maternal family history” or “paternal family history,” the side of the family was set as maternal or paternal.

(3) If there was an indicator (“maternal” or “paternal”) near a family member, the side of the family was determined by the indicator.

(4) Otherwise, the side of the family of a family member was set as “NA.”

4. Determining the living status score of family members following the work of Shi et al [17].

(1) Determining the scores of the properties “Alive” and “Healthy” of a family member through searching the keywords listed in Table 3 from the family member’s living status. If a living status entity contained some keywords listed in Table 2, we assigned its property scores with the corresponding scores; otherwise, both its alive score and healthy score were set as NA=1.

(2) The total living status score was determined according to the alive score and healthy score. For a relative with “Alive=Yes” and “Healthy=Yes,” for example, the living status score should be 4.

5. Determining the negation information of observations.

(1) Determining the negation information of an observation through searching keywords (no, never, not, none, negative, neither, nor, unremarkable, and deny) from the observation’s context. If the context of an observation contained a keyword mentioned above, we set its negation information as “Negated;” otherwise, it was set as “Non_Negated.”

(2) Reversing the negation information of an observation if there were specific phrases, such as “apart from” and “except for,” in the observation’s context. For example, the negation information of the observation entity “Meniere disease” in “there is no history of hearing loss apart from the father's history of Meniere disease” was set as “Non_Negated” rather than “Negated.”

**Table 3:** Keywords used to determine the properties “Alive” and “Healthy.”

Property	Keywords
Alive: Yes=2	Alive and living
Alive: No=0	Dead, die, deceased, death, died, stillborn, and passed away
Healthy: Yes=2	Good, health, without problems, healthy, and well

### Experimental Settings

The hyperparameters used in our experiments are listed in Table 4, and all other parameters were optimized in the validation set. The pretrained BERT model we used was [BERT-Base, Uncased] [24].

We first investigated our model in the following two settings: (1) a pipeline model that tackled unlabeled arc prediction and arc label prediction separately and (2) a joint model that tackled unlabeled arc prediction and arc label prediction simultaneously. The joint model predicated the arc and label of each token in our model jointly. The pipeline model first trained one model to predict the head of each token and then trained another model to predict the head of each token according to the result of the predicted head. Thereafter, we compared our model with the BERT-based model using the same architecture as that of the model by Shi et al [17], except that we used BERT instead of word embeddings in the input layer (denoted by BERT-2BiLSTM). Finally, we looked into the effect of the sentence representation based on CNN-BiLSTM on our model and the effect of different data sets on our model. The performance of all models for the two subtasks was measured by precision, recall, and F1 score (F1) as follows:

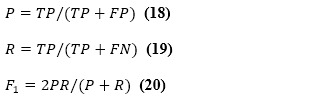



where TP denotes the number of true-positive samples, FP denotes the number of false-positive samples, and FN denotes the number of false-negative samples. We used the tool provided by the organizers [25] to calculate them. The tool accepted partial matching of the observations, for example, the recognized observation “diabetes” whose gold standard observation is “type 2 diabetes” was considered as a true-positive sample. The source code is available at GitHub [26].

**Table 4:** Major hyperparameters.

Parameter	Value
BiLSTM^a^ size	256
Arc MLP^b^ size	500
Label MLP size	100
BERT^c^ size	768
Char embedding size	25
CNN^d^ kernel size	(3, 4, 5)
Char-level CNN size	50
Dropout	0.5
Optimizer	Adam
Learning rate	2e-5
Batch size	32
Max epoch	100

^a^BiLSTM: Bidirectional Long Short Term Memory network.

^b^MLP: multilayer perceptron.

^c^BERT: Bidirectional Encoder Representation from Transformers.

^d^CNN: convolutional neural network.

## Results

As shown in Table 5, the performance of the model considering five types of entities was better than that considering three types of entities. The joint model considering five types of entities achieved the highest F1 score of 0.8823 on the NER subtask and 0.7048 on the RE subtask, which were higher than the values for the joint model considering three types of entities by 1.20% on the NER subtask and 1.87% on the RE subtask.

Compared to the pipeline model, the joint model performed better on both the NER and RE. For example, when considering five types of entities, the joint model outperformed the pipeline model by 1.21% in the F1 score on the NER subtask and 1.97% in the F1 score on the RE subtask. It indicated that error propagation was partially alleviated in our joint model. When considering five types of entities, the joint model achieved higher F1 scores than BERT-2BiLSTM on the NER subtask and RE subtask by 1.18% and 0.39%, respectively.

**Table 5:** Performance of different models.

Subtask	Model	Three types of entities				Five types of entities			
		Precision	Recall	F1 score	Precision		Recall	F1 score	
NER^a^	Pipeline	0.9254	0.8062	0.8617	0.9241		0.8223	0.8702	
NER	Joint	0.9012	0.8415	0.8703	0.9154		0.8514	0.8823	
NER	BERT^b^-2BiLSTM^c^	—^d^	—	—	0.9096		0.8347	0.8705	
RE^e^	Pipeline	0.7909	0.6005	0.6827	0.7895		0.6051	0.6851	
RE	Joint	0.7679	0.6200	0.6861	0.7717		0.6487	0.7048	
RE	BERT-2BiLSTM	—	—	—	0.7686		0.6441	0.7009	

^a^NER: named entity recognition.

^b^BERT: Bidirectional Encoder Representation from Transformers.

^c^BiLSTM: Bidirectional Long Short Term Memory network.

^d^Not available.

^e^RE: relation extraction.

The performance of our best model on each type of family member information and relation (except living status not provided in the test set) is listed in Table 6. On the NER subtask, our model performed better on observations than family members by 3.80% in terms of the F1 score. Among the three types of family members, our model achieved the highest F1 score of 0.8702 for maternal family member and the lowest F1 score of 0.8411 for paternal family member. On the RE subtask, the F1 score of our model on the family member-living status relation was nearly the same as that of our model on the family member-observation relation. Among the family member-observation relations, our model performed worse on the maternal-observation relation than the other two types of relations. Among the family member-living status relations, our model performed worse on the paternal-living status relation than the other two types of relations.

**Table 6:** Performance of the best model on each type of family member information.

Subtask	Type	Precision	Recall	F1 score
NER^a^	FM^b^: overall	0.8814	0.8386	0.8594
NER	FM: NA^c^	0.8699	0.8515	0.8606
NER	FM: maternal	0.9185	0.8267	0.8702
NER	FM: paternal	0.8738	0.8108	0.8411
NER	OB^d^	0.9385	0.8598	0.8974
NER	LS^e^	—^f^	—	—
NER	Overall	0.9154	0.8514	0.8823
RE^g^	FM-OB: overall	0.7843	0.6397	0.7047
RE	FM-OB: NA-OB	0.8595	0.6098	0.7134
RE	FM-OB: maternal-OB	0.7067	0.6601	0.6826
RE	FM-OB: paternal-OB	0.7077	0.7150	0.7113
RE	FM-LS: overall	0.7627	0.6553	0.7050
RE	FM-LS: NA-LS	0.7627	0.6553	0.7050
RE	FM-LS: maternal-LS	0.7108	0.7375	0.7239
RE	FM-LS: paternal-LS	0.6825	0.6825	0.6825
RE	Overall	0.7717	0.6487	0.7048

^a^NER: named entity recognition.

^b^FM: family member.

^c^NA: not applicable.

^d^OB: observation.

^e^LS: living status.

^f^Not available.

^g^RE: relation extraction.

As shown in Table 7, without using the additional data for BioCreative/OHNLP 2018, our model considering five types of entities achieved an F1 score of 0.8648 on the NER subtask and 0.6612 on the RE subtask (the F1 score was significantly reduced both on the NER subtask and RE subtask), showing the importance of the data.

**Table 7:** Performance of our model with different data.

Subtask	Data set	Three types of entities				Five types of entities		
		Precision	Recall	F1 score	Precision		Recall	F1 score
NER^a^	2019	0.8767	0.8409	0.8584	0.8847		0.8458	0.8648
NER	2018+2019^b^	—^c^	—	—	0.9154		0.8372	0.8745
NER	2018+2019^d^	0.9012	0.8415	0.8703	0.9154		0.8514	0.8823
RE^e^	2019	0.7240	0.5973	0.6545	0.7270		0.6064	0.6612
RE	2018+2019^b^	—	—	—	0.7459		0.6265	0.6810
RE	2018+2019^d^	0.7679	0.6200	0.6861	0.7717		0.6487	0.7048

^a^NER: named entity recognition.

^b^2018+2019: the challenge submission performances of our model.

^c^Not available.

^d^2018+2019: the performances of our best model after challenge.

^e^RE: relation extraction.

## Discussion

### Effect of Sentence Representation

In order to investigate the effect of sentence representation based on CNN-BiLSTM on our model, we evaluated the model without using the representation and obtained an F1 score of 0.8802 on the NER subtask and an F1 score of 0.7059 on the RE subtask when considering five types of entities. The sentence representation based on CNN-BiLSTM can bring improvement in the NER subtask, but a little loss in the RE subtask. Possibly, we can only share BERT on NER and RE for further improvement.

### Impact of Different Decoders on the NER Subtask

Traditional approaches regarded the NER task as a sequence labeling task, in which each token was assigned with a combined label of entity boundary and type. The entity boundaries were represented by the BIO schema, where “B” indicates the beginning of an entity, “I” indicates the inside of an entity, and “O” indicates the outside of an entity. Using a graph schema, we can also convert NER into a graph in the following way: (1) connect all tokens with “ROOT,” that is, the heads of all tokens are set to 0 and (2) set the label of the nonentity token to “NULL,” set the label of the last token in the entity to the entity type, and set the label of the remaining token in the entity to “app.”

We compared different decoders, that is, CRF for sequence labeling, biaffine for NER only (biaffine-NER), and biaffine for joint NER and RE (biaffine-Joint). As shown in Table 8, the performance of biaffine-NER was slightly better than that of CRF, while biaffine-Joint was considerably better than the other two models. Although the head prediction was not directly related to the NER task, the arcs of different types among tokens provided global information that was beneficial to the NER task.

**Table 8:** Comparison of different decoders on the named entity recognition subtask.

Decoder	Three types of entities			Five types of entities		
	Precision	Recall	F1 score	Precision	Recall	F1 score
CRF^a^	0.8989	0.8316	0.8639	0.9070	0.8390	0.8717
Biaffine-NER^b^	0.9001	0.8310	0.8641	0.8895	0.8570	0.8729
Biaffine-Joint	0.9012	0.8415	0.8703	0.9154	0.8514	0.8823

^a^CRF: conditional random field.

^b^NER: named entity recognition.

### Error Analysis

We performed error analysis on our model considering five types of entities in the development data set. In the case of the NER subtask, 88.24% of errors were boundary errors because of wrong “app” arc prediction, while the remaining 11.76% of errors were type errors that have a correct boundary but wrong entity type. For example, in the sentence “The paternal grandmother, age 53, has wind sucking attributed to not having intestinal during her life,” the paternal entity “grandmother” with the observation entity “wind sucking” was wrongly recognized as a family member entity. In the RE subtask, all errors were caused by incorrect entities. For example, in the sentence “The patient’s father is 43 years old and healthy. His father is 72 years old and was diagnosed with esophageal cancer at age 70,” the family member entity “grandfather” with the observation entity “esophageal cancer” was wrongly extracted as the family member entity “father” with the observation entity “esophageal cancer” as our model could not understand that “his” refers to “the patient’s father,” which needs strong indirect relative reasoning.

### Limitations and Future Work

The rule-based postprocessing module in our system cannot handle all cases properly, as shown by the example in the error analysis section. In future work, we will try to solve indirect relative reasoning for further improvement.

### Conclusions

In this study, we proposed a novel graph-based model with biaffine attention, where a graph-based schema was design to represent entities and relations regarding family history in a unified way and deep biaffine attention was adopted to extract the entities and relations from clinical text. Our system based on the proposed model achieved the highest F1 score of the challenge to date.

## Abbreviations

BERT: Bidirectional Encoder Representation from Transformers
BiLSTM: Bidirectional Long Short Term Memory
CNN: convolutional neural network
CRF: conditional random field
EHR: electronic health record
NER: named entity recognition
NLP: natural language processing
RE: relation extraction
